# Host-response transcriptional biomarkers accurately discriminate bacterial and viral infections of global relevance

**DOI:** 10.1038/s41598-023-49734-6

**Published:** 2023-12-18

**Authors:** Emily R. Ko, Megan E. Reller, L. Gayani Tillekeratne, Champica K. Bodinayake, Cameron Miller, Thomas W. Burke, Ricardo Henao, Micah T. McClain, Sunil Suchindran, Bradly Nicholson, Adam Blatt, Elizabeth Petzold, Ephraim L. Tsalik, Ajith Nagahawatte, Vasantha Devasiri, Matthew P. Rubach, Venance P. Maro, Bingileki F. Lwezaula, Wasantha Kodikara-Arachichi, Ruvini Kurukulasooriya, Aruna D. De Silva, Danielle V. Clark, Kevin L. Schully, Deng Madut, J. Stephen Dumler, Cecilia Kato, Renee Galloway, John A. Crump, Geoffrey S. Ginsburg, Timothy D. Minogue, Christopher W. Woods

**Affiliations:** 1 Division of General Internal Medicine, Department of Medicine, Duke Regional Hospital, Duke University Health System, Duke University School of Medicine, 3643 N. Roxboro St., Durham, NC 27704 USA; 2 Division of Infectious Diseases, Department of Medicine, Duke University School of Medicine, Durham, NC USA; 3 Durham Veterans Affairs Health Care System, Durham, NC USA; 4 Duke Global Health Institute, Duke University, Durham, NC USA; 5 Department of Medicine, Faculty of Medicine, University of Ruhuna, Galle, Sri Lanka; 6 Clinical Research Unit, Department of Medicine, Duke University School of Medicine, Durham, NC USA; 7 Department of Biostatistics and Informatics, Duke University, Durham, NC USA; 8 Institute for Medical Research, Durham, NC USA; 9 Division of Pediatric Infectious Diseases, Department of Pediatrics, Duke University School of Medicine, Durham, NC USA; 10 Danaher Diagnostics, Washington, DC USA; 11 Department of Microbiology, Faculty of Medicine, University of Ruhuna, Galle, Sri Lanka; 12 Programme in Emerging Infectious Diseases, Duke-National University of Singapore, Singapore, Singapore; 13 Kilimanjaro Christian Medical Center, Moshi, Tanzania; 14 Kilimanjaro Christian Medical University College, Moshi, Tanzania; 15 Maswenzi Regional Referral Hospital, Moshi, Tanzania; 16 General Sir John Kotelawala Defence University, Colombo, Sri Lanka; 17 The Henry M. Jackson Foundation for the Advancement of Military Medicine, Bethesda, MD USA; 18 Austere Environments Consortium for Enhanced Sepsis Outcomes (ACESO), Biological Defense Research Directorate, Naval Medical Research Center-Frederick, Ft. Detrick, MD USA; 19 Joint Departments of Pathology, School of Medicine, Uniformed Services University, Bethesda, MD USA; 20 Centers for Disease Control and Prevention, National Center for Emerging Zoonotic Infectious Diseases, Atlanta, USA; 21 Centre for International Health, University of Otago, Dunedin, New Zealand; 22 Department of Medicine, Duke University School of Medicine, Durham, NC USA; 23 National Institute of Health, Bethesda, MD USA; 24 Diagnostic Systems Division, USAMRIID, Fort Detrick, Frederick, MD USA

**Keywords:** Computational biology and bioinformatics, Microbiology, Biomarkers

## Abstract

Diagnostic limitations challenge management of clinically indistinguishable acute infectious illness globally. Gene expression classification models show great promise distinguishing causes of fever. We generated transcriptional data for a 294-participant (USA, Sri Lanka) discovery cohort with adjudicated viral or bacterial infections of diverse etiology or non-infectious disease mimics. We then derived and cross-validated gene expression classifiers including: 1) a single model to distinguish bacterial vs. viral (Global Fever-Bacterial/Viral [GF-B/V]) and 2) a two-model system to discriminate bacterial and viral in the context of noninfection (Global Fever-Bacterial/Viral/Non-infectious [GF-B/V/N]). We then translated to a multiplex RT-PCR assay and independent validation involved 101 participants (USA, Sri Lanka, Australia, Cambodia, Tanzania). The GF-B/V model discriminated bacterial from viral infection in the discovery cohort an area under the receiver operator curve (AUROC) of 0.93. Validation in an independent cohort demonstrated the GF-B/V model had an AUROC of 0.84 (95% CI 0.76–0.90) with overall accuracy of 81.6% (95% CI 72.7–88.5). Performance did not vary with age, demographics, or site. Host transcriptional response diagnostics distinguish bacterial and viral illness across global sites with diverse endemic pathogens.

## Introduction

Infectious diseases are leading causes of morbidity and mortality worldwide^1–3^. The toll is greatest in low- and middle-income countries (LMIC), where infections are frequently caused by pathogens that cannot be identified when patients present with fever and resources for testing and treatment are limited. High rates of malnutrition and HIV exacerbate the problem by contributing to increased susceptibility to infection and diversity of pathogens^4–8^. Without sensitive and specific point-of-care diagnostics to rapidly confirm or refute multiple etiologies of fever, bacterial infections remain untreated and viral infections are treated with antibiotics unnecessarily. The result has been unprecedented inappropriate antibiotic use and associated increasing antimicrobial resistance^9–17^. The World Health Organization estimates that by 2050 antimicrobial resistance will lead to 10 million lives lost and cost 100 trillion USD per year, leading to an urgent called for new diagnostic assays and approaches to combat the problem^18^.

Host-response transcription patterns could fill this diagnostic gap by distinguishing between bacterial and viral etiologies early^19–27^, including before symptoms, to limit spread and guide resource allocation^28–30^. Gene expression classification models have shown great promise for the classification of causes of fever in high-income countries (HIC)^31,32^ with progress extending to atypical pathogens present in LMIC^20,26,33–35^. These multi-analyte gene expression models can be translated to rapid diagnotic platforms that inform clinical care^32–34,36^. In this study, we generated host response biomarkers for the varied etiologies of suspected infection important worldwide, translated them to a quantitative RT-PCR multiplex platform, and validated them in a globally diverse independent cohort.

## Methods

### Global fever discovery and validation cohorts

Participants were prospectively enrolled within 48 h of presentation to academic hospitals in the USA^25,37–39^, Sri Lanka^40–43^, Tanzania^44,45^, Cambodia^46–48^, and Australia (Supplemental Table 1). Samples from participants were stored in a Duke University international biorepository and selected for analysis if they met inclusion critieria for suspected infection defined as: 1) a qualifying vital sign or lab abnormalities (fever ≥ 38.0 °C or ≤ 36 °C, heart rate ≥ 90, respiratory rate ≥ 20, and/or white blood cell count ≥ 12 (cells × 10^9^L), 2) clinical symptoms consistent with acute infection, and 3) adjudicated as meeting bacterial, viral, or noninfectious case definitions (Supplemental Table 2). A committee inclusive of clinical and statistical teams made final cohort selections, ensuring adequate balance among demographic and infectious phenotypes. The discovery cohort included 294 participants presenting to academic hospitals in the USA (n = 152) or Sri Lanka (n = 142). The validation cohort included 101 participants enrolled in the USA (n = 19), Sri Lanka (n = 53), Tanzania (n = 15), Cambodia (n = 10), and Australia (n = 4).

**Table 1:** Demographics and participant characteristics of discovery and validation cohort.

Discovery cohort	USA				Sri Lanka			
	Total (n = 294)	Bacterial (n = 42)	Viral (n = 43)	Noninfectious (n = 67)	Bacterial (n = 60)	Viral (n = 82)		
Age, median (IQR)	48 (31, 61)	58 (49, 66)	50 (28, 66)	54 (42, 66)	41 (31, 50)	34 (23, 51)		
Sex, n (%)								
Male	155 (52.7)	28 (66.7)	14 (32.6)	30 (44.7)	39 (66.7)	38 (51.2)		
Female	139 (47.3)	14 (33.3)	29 (67.4)	37 (55.3)	21 (33.3)	44 (48.8)		
Race, n (%)								
Hispanic	142 (48.3)	38 (90.5)	39 (93.0)	65 (97.0)	–	–		
Non-Hispanic	4 (1.4)	2 (4.8)	1 (2.3)	1 (1.5)	–	–		
	5 (1.7)	2 (4.7)	2 (4.6)	1 (1.5)	–	–		
Ethnicity, n (%)								
White	87 (29.5)	27 (64.3)	25 (58.1)	35 (52.2)	–	–		
Black	56 (19.0)	13 (31.0)	14 (32.6)	29 (43.3)	–	–		
Asian/S. Asian	144 (49.0)	0 (0.0)	0 (0.0)	2 (4.7)	60 (100)	82 (100)		
American In/Alaska Na	5 (1.7)	2 (4.8)	1 (2.3)	2 (3.0)	–	–		
Hawaiian/Pacific Is	1 (0.3)	0 (0.0)	1 (2.3)	0 (0.0)	–	–		
Other/unknown	1 (0.3)	0 (0.0)	0 (0.0)	1 (1.5)	–	–		
Duration ill, median days (IQR)	4 (3, 6)	3 (2, 5.5)	4 (2. 5, 5.5)	3 (2, 11)	5 (3, 8)	4 (3, 5)		
Hospital length of stay, median days (IQR)	4 (2, 6)	6 (3, 9)	0 (0, 15)	2 (1, 6)	5 (4, 6)	4 (3, 6)		
Intensive care, n (%)	26 (8.8)	11 (26.2)	3 (7.0)	11 (16.4)	1 (1.7)	0 (0.0)		
Mech. ventilation, n (%)	14 (4.8)	6 (14.3)	1 (2.3)	6 (9.0)	1 (1.7)	0 (0.0)		
Mortality, n (%)	12 (4.0)	5 (11.9)	0 (0.0)	6 (9.0)	0 (0.0)	1 (1.2)		
Pathogens	–	11 *S. aureus*	17 FluA	NA	30 *Leptospira* spp.	43 Dengue		
		14 *S. pneumo*	12 FluB		27 *Rickettsia* spp.	23 FluA		
		9* E. coli*	14 RSV		3 *C. burnetii*	16 FluB		
		9 K*. pneumo*						

Validation cohort	USA			Sri Lanka		Cambodia	Tanzania	Australia
	Total (n = 101)	Bacterial (n = 3)	Viral (n = 16)	Bacterial n = 24)	Viral (n = 29)	Bacterial (n = 10)	Bacterial (n = 15)	Viral (n = 4)
Age, median (IQR)	36.5 (27.8, 54.3)	61 (60.5, 73.5)	49 (29, 60)	37 (29.5, 58)	30 (25, 47)	51 (36.3, 58.8)	34 (30.5, 41.5)	27 (27, 35.5)
Sex, n (%)								
Male	55 (54.5)	3 (100)	5 (31.3)	14 (58.3)	21 (72.4)	5 (50.0)	6 (40.0)	1 (25.0)
Female	46 (45.5)	0 (0.0)	11 (68.8)	10 (41.7)	8 (27.6)	5 (50.0)	9 (60.0)	3 (75.0)
Race, n (%)								
Hispanic	–	3 (100)	16 (100)	–	–	–	–	4 (100)
Non-Hispanic	–	0 (0.0)	0 (0.0)	–	–	–	–	0 (0.0)
Unknown	–	0 (0.0)	0 (0.0)	–	–	–	–	0 (0.0)
Ethnicity, n (%)								
White	12 (11.9)	2 (66.7)	6 (37.5)	–	–	–	–	4 (100)
Black	26 (25.7)	1 (33.3)	10 (62.5)	–	–	–	15 (100)	0 (0.0)
Asian/S. Asian	63 (62.4)	0 (0.0)	0 (0.0)	24 (100)	29 (100)	10 (100)	–	0 (0.0)
Pathogens		1 *S. aureus*	2 Dengue	15 *Leptospira* spp.	18 Dengue	*10 B. pseudomallei*	3 *Brucella* spp.	4 FluA
		1 *VGS*	4 FluA	9 *Rickettsia* spp.	7 FluA		2 *Rickettsia* spp*.*	
		1 *P. aerug*	2 RSV		4 HRV		10 *C. burnetii*	
			2 HRV					
			3 Paraflu					
			3 hMPV					

*Days ill* number of days ill prior to presentation, *ICU* Intensive care unit, *Mech. Ventilation* Invasive Mechanical Ventilation, *NA* not applicable, *S. aureus Staphylococus aurus*, *S. pneumo Steptococcus pneumoniae*, *E. coli Escherichia coli*, *K. pneumo Klebsiella pneumoniae*, *FluA* Influenza A, *FluB* Influenza B, *RSV* Respiratory Syncitial Virus, *C. burnetii Coxiella burnetii*, *VGS* Viridians group Streptococcus, *P. aerug Pseudomonas aeruginosa*, *HRV* Human Rhinovirus, *Paraflu* ParaInfluenza, *hMPV.* human Metapneumovirus, *B. pseudomallei* Burkholderia pseudomallei.

**Table 2:** Differential expression of genes upregulated at least tenfold in bacterial versus viral illness.

Functional category	Genes
Upregulated in bacterial infection	
Acid/base equilibrium	CA4
Acute phase reactants	ALPL, C4BPA, HP, HPR, ORM1, ORM2
Antimicrobial killing	ARG1, PGLYRP1, PI3, S100A12, SLPI
Apoptosis/development	KREMEN1
Cell division	SPATC1
Cell migration	ITGA7
Cell motility	CFAP126
Epigenetics	KDM5D
Extracellular matrix integrity	ADAMTS2, PCOLCE2
Heavy metal binding	MT1H
Immune regulation (cell surface receptors)	CD177, CD300LD, VSIG4
Innate immune response	IL1R2
Metabolism	OLAH, SLC51A, VNN1
Protein degradation	MMP8, PGA4
Protein processing/sorting	AP3B2, FAM20A, GALNT14, ZDHHC19
Signal transduction	BMX, NECAB1, RCVRN
Transcription	KLF14
Translation	EIF1AY
Upregulated in viral infection	
Amino acid metabolism	IL4I1, SDS
Antimicrobial killing	DEFB1
Apoptosis	BCL2L14
Autophagy	RUFY4
Cell activation receptors	LY6E
Cell–cell interactions	AGRN, DSP, SIGLEC1
Cell differentiation/growth	AXL, EPHB2
Cell motility	DZIP1L, TTC21A
Cell structure/junctional	JUP, KRT5, NEXN, OTOF, SAMD4A
Electrochemical gradiant	NKAIN1
Exocytosis	EXOC3L1
Interferon response/chemokines/cytokines	DDX60, HERC5, HERC6, IFI27, IFI44, IFIT1, IFIT2, IFIT3, ISG15, LAMP3, MX1, NRIR, OAS1, OAS2, OAS3, OASL, RSAD2, USP18, CCL2, CCL8, CXCL10, CXCL11, FPR3, LIP
Intracellular trafficking	FBX039, RABGAP1L, RIN2
Mitochondrial DNA synthesis	CMPK2
Pattern recognition receptors	CLEC4F, TLR3
Transcription	HES4, HESX1, ZNF684
Non-coding or poorly characterized/unknown	ALMS1P1, ERICH3, HSPB9, KCTD14, LINC00487, LOC100133669, LOC101927027, LOC105369192, SPATS2L, TMEM252, TMEM255A, XIST

Genes upregulated in bacterial disease are conversely downregulated in viral disease and vice versa.

### Samples and etiologic testing

Blood was collected at enrollment in PAXgene RNA tubes (QIAGEN) at all sites. Sera were collected at both enrollment (acute phase) and 2–6 week follow-up (convalescent phase) in Sri Lanka and Tanzania. Naso-pharyngeal swabs were collected at enrollment in the USA, Sri Lanka, and Australia. All samples were processed according by standardized protocols, stored at − 70 °C, and shipped on dry ice.

Etiologic testing was performed using reference standard methods to confirm or refute possible bacterial and viral causes of suspected infection endemic to the region. Blood culture and/or urine antigen tests performed as part of clinical care confirmed bacteremia for USA subjects. Bacterial isolates and urine collected in Cambodia confirmed *Burkholderia pseudomallei* by blood culture, sputum culture, and/or urine antigen testing^47,49^. For participants enrolled in Sri Lanka and Tanzania, bacterial zoonoses were confirmed by a ≥ fourfold rise in titer of microscopic agglutination testing for *Leptospira* spp. and *Brucella* spp.^44,45^, or indirect immunofluorescence assay for *Rickettsia* spp. (Spotted Fever Group, Typhus Group, and *Orientia tsutsugamushi*) and *Coxiella burnetii*, and/or by polymerase chain reaction (PCR) in a USA reference laboratory. For participants enrolled in the USA and Sri Lanka, respiratory viral infections were confirmed by PCR on nasopharyngeal samples (Luminex Integrated System NxTAG Respiratory Pathogen Panel; Luminex Corporation; Austin, TX)^50^. For those enrolled in Sri Lanka, acute dengue was confirmed by fourfold rise in antibody titer, viral isolation, and/or PCR at a reference laboratory^41,51^. The Tanzania study performed blood culture and/or blood smears for malarial pathogens.

## Reference standard adjudication of etiology

Phenotypic adjudication of bacterial, viral, or noninfectious etiology independent of cohort selection (described above) was performed by a panel of ≥ 2 physicians who reviewed all available microbiologic data, de-identified clinical data extracted from case report forms (international), or the full medical records (USA) (Supplemental Table 2). Participants known to have malaria by blood smear were excluded due to insufficient frequency required to generate a parasitic classifier. Non-infectious cases had supportive clinical and radiographic data along with negative testing for infectious etiologies. Infectious cases were defined by positive etiology testing and supportive clinical data. Participants included from Tanzania had confirmed bacterial etiologic testing, but did not undergo testing for viral co-infection because dengue testing and respiratory viral swab were not available as part of this study (Supplemental Table 1).

### Generation and normalization of transcriptomic data

Total RNA was extracted from whole blood collected and stored at − 70 °C in PAXgene Blood RNA tubes using the PAXgene miRNA Extraction Kit (QIAGEN) according to manufacturer’s instructions. RNA yield and integrity were assessed using NanoDrop ND-2000 spectrophotometer (ThermoFisher Scientific, Wilmington, DE) and 2100 Bioanalyzer with RNA 6000 Nano kit (Agilent Technologies, Santa Clara, CA), respectively. All RNA was purified under BSL3 conditions by approved protocols at Duke Regional Biocontainment Laboratory, except *B. pseudomallei* mRNA isolated under BSL4 conditions by standard procedures at the Navel Medical Research Laboratory.

RNA sequencing was performed at EAGenomics/Q2 Labs (Durham, NC) for 183 samples and the Duke Sequencing and Genomic Techologies Facility for 111 samples. Library preparation resulted in selected poly-A mRNA for sequencing using GlobinClear RNA Reduction (Invitrogen) and TruSeq Stranded mRNA Library Kit (Illumina) for the EA Genomics/Q2 Labs batch, and NuGEN Universal Plus mRNA-Seq Library Prep Kit with AnyDeplete Globin depletion (NuGEN/Tecan) for the Duke Sequencing Facility batch. Sequencing libraries were sequenced on Illumina HiSeq 2500 instrument (EA Genomics/Q2 Labs) or NovaSeq 6000 instrument (Duke Sequencing Facility) with 50 bp paired-end reads and target of > 40 million reads per sample, including crossover of 24 samples between the two batches to allow for quality control and batch corrections.

### Nanostring multiplex transcript detection platform

Quantitative RT-PCR assays for genes in both the Global Fever Bacterial/Viral (GF-B/V) and Global Fever-Bacterial/Viral/Noninfectious (GF-B/V/N) models were developed using the NanoString platform. Total RNA (100 ng) from each participants was analyzed using a NanoString nCounter XT custom transcriptional response probe panel (NanoString Technologies, Seattle, WA). Nanostring assay processing was performed by the Duke Microbiome Core Facility according to manufacturer instructions.

### Statistical analysis

We used Limma-voom modeling to obtain differential expression of transcripts ≥ tenfold in bacteria versus virus infected participants with an adjusted p-value < 0.01 in the discovery cohort. A cutoff of ≥ tenfold was used to identify the most highly differentially expressed genes. A significance threshold of 5% false discovery rate (FDR) was used. Pathway analysis used Database for Annotation, Visualization and Integrated Discovery (DAVID) and ENRICHr programs to create broad functional groups. Transcripts that did not fit into well-defined ontologic clusters were categorized by literature review.

**Figure 1:**
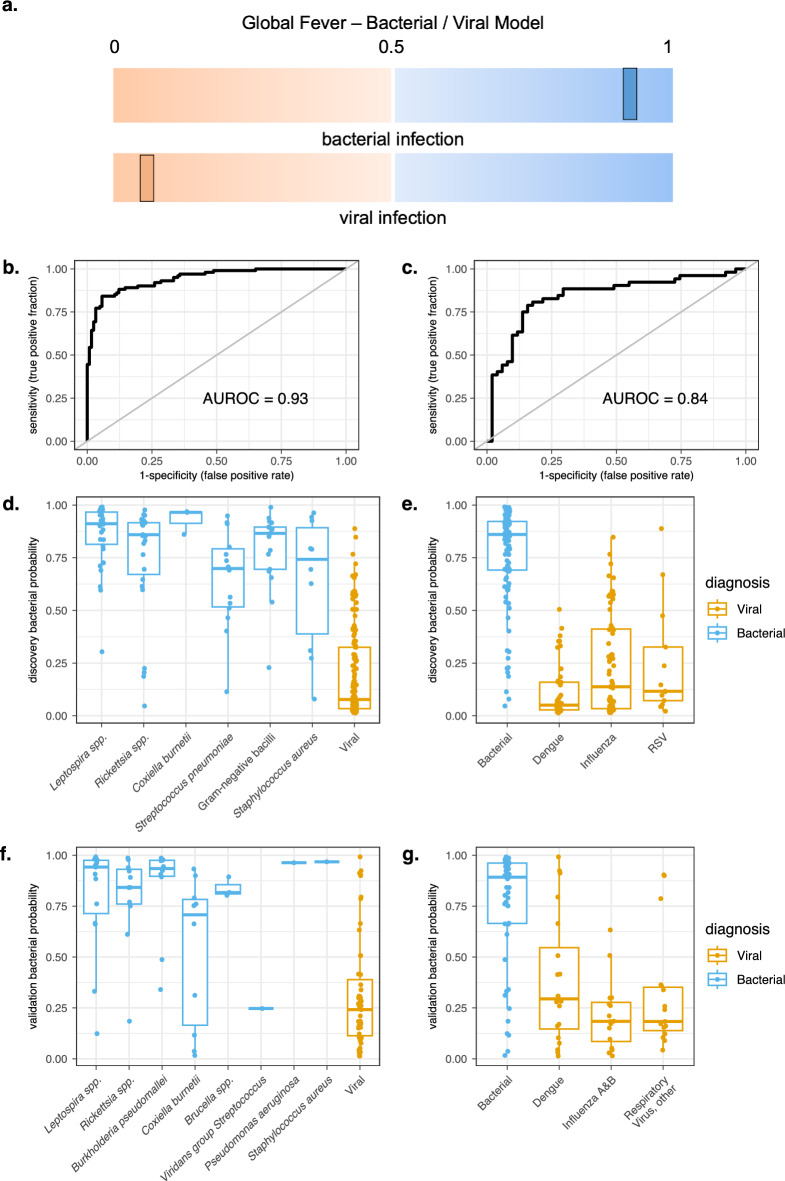
Performance of GF-B/V model to classify bacterial and viral disease in a global cohort. (**A**) A binary model (GF-B/V) provides a single score that discriminates bacterial from viral infection. High probabilities closer to 1 are associated with bacterial infection and low probabilities closer to 0 indicate viral infection. (**B**) AUROC curve of the discovery cohort (RNA sequencing) for GF-B/V model. (**C**) AUROC curve of the validation cohort (NanoString platform) for GF-B/V model. (**D**) Predicted probabilities for the GF-B/V model in the discovery cohort for bacterial pathogens (blue) compared to viral pathogens (orange) using RNA sequencing. (**E**) Predicted probabilities for the GF-B/V model in the discovery cohort for bacterial pathogens (blue) compared to viral pathogens (orange) using NanoString assay. Bacterial abbreviations: Gram negative bacilli = *Escherichia coli*, *Klebsiella pneumoniae*, Rickettsia spp. = Spotted fever group, Typhus group, *Orientia tsutsugamushi*. Viral abbreviations: Other Resp. Virus = human Rhinovirus, Parainfluenza, human Metapneumovirus, Respiratory Syncytial Virus.

To develop predictive models, the discovery cohort included Duke and Sri Lanka participants because these sites had similar extensive phenotypic analysis for both bacterial and viral pathogens and adequate populations of at least two of the phenotypes classes. We developed a simple binary GF-B/V model including only participants with bacterial or viral infection (Fig. 1A). Since fever or suspected infections may be neither bacterial nor viral, we incorporated participants with non-infectious illness as a control group in a second modeling approach (GF-B/V/N). The GF-B/V/N model used two binary predictive classifiers for discrimination: bacterial vs. non-bacterial (viral or non-infectious), and viral vs. non-viral (bacterial or non-infectious). The categorization of bacterial or viral illness by the GF-B/V/N test is made for each participant by comparing the probabilities of each binary classifier (Supplemental Fig. 1A). High-confidence noninfectious samples were only available from the USA, but there were no significant difference in expression of control house-keeping genes that would suggest a site specific or confounding affect.

Standard quality control and principal component analysis was performed and ensured there were no site dependent effects or inappropriate clustering of the data. We then conducted supervised regularized regression (Least Absolute Shrinkage and Selection Operator [LASSO]) analysis of the entire transcriptome. Nested, repeated (500 repeats) fivefold cross-validation was performed to estimate predicted probabilities. All model-building steps were performed on training data only to maintain unbiased estimates generated on the test fold. Predicted probabilities were utilized to estimate area under the receiver operating curve (AUROC) and ROC01 method was used to select a cutoff to estimate accuracy and characterize performance. Use of 500 sets of predictions for the discovery cohort limited calculation of predicted confidence intervals by the standard approach^52^, but was more representative of model development.

The validation cohort was designed to represent a more typical global population; thus, sites representative of a single class or with less extensive phenotyping were included. To generate NanoString nCounter assays, we expanded feature prediction to include correlated transcripts that can substitute for one another with respect to class prediction (bacterial, viral, or noninfectious). Feature selection was performed using elastic net regression and the selection frequency across resampling iterations measured variable importance. Characterizing performance in a targeted validation study required selecting 263 transcripts (Supplementary Table 3). Endogenous control transcripts (TRAP1, DECR1, TBP, and PPIB) were incorporated to normalize for differences in sample input and correct for technical variability. A model was trained on the NanoString data using 91 participants from the discovery cohort (47 bacterial, 34 viral, 10 noninfectious), accommodating known positive control normalization to reduce technical variability and allow background subtraction using negative controls. Discovery cohort participants selected for model training on NanoString prioritized three goals in the following order: 1) balance of infectious etiologies and phenotypes, 2) robust performance in the discovery models, and 3) representation from diverse geographic regions and pathogens. Noninfectious samples were not incorporated into the validation cohort due to availability of unique specimens and a desire to incorporate increased infectious etiologies. The NanoString GF-B/V and GF-B/V/N models were then fixed and applied to the independent validation cohort.

Confidence intervals were calculated using the epiR package in R. exact binomial for the sensitivity, specificity, and model accuracy^53^. The approach of Simel et al. was used to calculate confidence intervals for the positive and negative likelihood ratios^54^. Confidence intervals for the validation AUROCs were calculated using the method of DeLong^52^. A confidence interval for the overall accuracy of the GF-B/V/N model for the discovery cohort was estimated by taking 10,000 bootstrapped samples. We used the nonparametric Mood’s median test to calculate the p-value estimating the differences in median ages and to evaluate whether the proportion of women in bacterial samples was different than non-bacterial samples.

### Ethical approval

Prospective collection of specimens and data after written informed consent by subjects or their legally authorized representatives, and assent was obtained for minors less than 18 years old. Studies were approved by Institutional Review Boards of Duke University Health System, Faculty of Medicine, University of Ruhuna, Johns Hopkins University, Naval Medical Research Center, Kilimanjaro Christian Medical Center Research Ethics Committee, Tanzania National Institute for Medical Research National Research Ethics Coordinating Committee, University of Otago Human Ethics Committee (Health), and the USA CDC. This study used deidentified specimens and clinical data, and was approved by Duke University Health System (Durham, NC) Institutional Revew Board (Duke IRB Pro00072857). All research was conducted in accordance with the Declaration of Helsinki.

## Results

### Participants and pathogens

The discovery cohort consisted of participants from the USA and Sri Lanka with median age 48 years (IQR 31–61; range 10–86 years), 48% female, 1.4% Hispanic, 28.5% White, 19% Black/African American, 49.5% Asian/South Asian (Table 1). The median age of the USA cohort was higher than the Sri Lankan cohort (54 years [(IQR 42–66] vs. 37 years [IQR 26–51], p = 0.51), although this was not statistically significant. Those with bacterial infections were more likely to be male (p = 0.001), but this was not site or pathogen specific. USA participants had more severe illness (intensive care, 16.4% [n = 25/152], mechanical ventilation, 8.5% [n = 13/152], and mortality 7.2% [n = 11/152]) than those in Sri Lanka (intensive care, 0.7% [n = 1/142], mechanical ventilation, 0.7% [n = 1/142], and mortality, 0.7% [n = 1/142]). However, determining severity of illness between internationally diverse clinical settings, types of infection, and standards of care may be misleading. Chronic HIV was low across the total cohort (3 USA in discovery cohort, 3 Tanzania in validation cohort), and although HIV status was not collected for Sri Lanka the incidence in the country is < 0.01%.

The discovery cohort included 102 participants with bacterial (42 with bloodstream infections and 60 bacterial zoonoses), 125 with viral (82 respiratory, 43 dengue), and 67 with non-infectious illness (e.g., pulmonary embolus, congestive heart failure, COPD/Asthma, cancer, autoimmune disorders). The validation cohort had 101 participants (52 bacterial, 49 viral) and represented a wider range of demographics, geographic locations (USA, Sri Lanka, Tanzania, Cambodia, and Australia), and pathogens (Table 1). Patients with non-infectious illness were not analyzed in the validation cohort.

### Differential gene expression of global pathogens

To identify differentially expressioned genes, we employed a conservative approach, using a 5% FDR and a ≥ tenfold change in expression. We identified 38 unique genes increased at least tenfold in participants with bacterial illness, and these were divided into 18 primary clusters (Table 2). Transcripts corresponded to known pathways for acute phase reactants, antimicrobial killing, innate immunity, and immune response. Similarly, we identified 65 unique genes associated with increased expression by tenfold or greater in viral infection, and these were divided into 17 primary clusters (Table 2) primarily corresponding to interferon response and chemokine/cytokine pathways.

### Bacterial versus viral classification: a simple binary model

We conducted predictive analysis to develop a binary model (Fig. 1A) using the entire transcriptome from the discovery cohort. The Global Fever-Bacterial/Viral (GF-B/V) model classified bacterial from viral disease with high accuracy when internally validated using fivefold cross-validation: AUROC of 0.93 (Fig. 1B), sensitivity of 84.2% (95% CI 75.6–90.7), specificity of 94.7% (95% CI 88.6–97.7), and overall accuracy of 89.7% (95% CI 85.0–93.4). Additional performance characteristics are shown in Table 3. The model demonstrated similar performance after stratifying for specific pathogen (Fig. 1D), site, age, sex, or race (Supplemental Fig. 2).

**Table 3:** Performance characteristics for Global Fever classifier models for acute bacterial and viral infection.

Cohort	Comparison	Sensitivity, % (95% CI)	Specificity, % (95% CI)	Model accuracy, % (95% CI)	Positive likelihood ratio (95% CI)	Negative likelihood ratio (95% CI)
Global fever bacterial/viral model (GF-B/V)						
Discovery	Bacterial versus viral	84.2 (75.6–90.7)	94.7 (88.6–97.7)	89.7 (85.0–93.4)	14.7 (7.2–30.5)	0.2 (0.1–0.3)
Validation	Bacterial versus viral	78.8 (65.3–88.9)	84.3 (71.4–93.0)	81.6 (72.7–88.5)	5.0 (2.6–9.6)	0.3 (0.1–0.4)
Global fever bacterial/viral/noninfectious model (GF-B/V/N)						
Discovery	Bacterial versus nonbacterial	87.7 (79.0–89.8)	84.2 (78.2–89.1)	85.2 (80.6–89.1)	5.5 (3.9–7.7)	0.2 (0.1–0.3)
	Viral versus nonviral	83.7 (76.0–89.8)	81.5 (74.8–87.1)	82.5 (77.6–86.7)	4.5 (3.3–6.3)	0.2 (0.1–0.3)
Validation	Bacterial versus nonbacterial	82.7 (69.7–91.8)	80.4 (66.9–90.2)	81.6 (72.7–88.5)	4.2 (2.4–7.5)	0.2 (0.1–0.4)
	Viral versus nonviral	76.5 (62.5–87.2)	80.8 (67.5–90.4)	78.6 (69.5–86.1)	4.0 (2.237.1)	0.3 (0.2–0.5)

The top of the table provides performace characteristics for the GF-B/V model and the bottom of the table shows performance of the GF-B/V/N model. In the discovery cohort, performance characteristics are calculated using nested cross validation on the original RNA sequencing data. In the validation cohort, the model is fixed and applied to NanoString data of an independent bacterial and viral cohort. Positive and negative predictive value requires knowledge of prevalence in the community which is not known for global infections. Thus, these could not be calculated.

To independently validate this model using a quantitative RT-PCR system that more closely approximates a clinical assay, we used the NanoString system to measure expression levels of 27 highly predictive genes (Supplemental Table 4A). After training a classification model on subjects from the discovery cohort, the model and its parameters were fixed and applied to the validation cohort. We incorporated both pathogen and geographic diverisity (Table 1). For the discrimination of bacterial and viral infection, the GF-B/V model an AUROC of 0.84 (95% CI 0.76–0.9) (Fig. 1C), sensitivity of 78.8% (95% CI 65.3–88.9), specificity of 84.3% (95% CI 71.4–93.0), and overall accuracy of 81.6% (95% CI 72.7–88.5) with additional performance characteristics reported (Table 3). Additionally, GF-B/V discriminated difficult-to-diagnose bacterial zoonotic pathogens not included in the discovery cohort, such as spotted fever group rickettsiae, *B. pseudomallei*, and *Brucella* spp. (Fig. 1E).

### Classification of bacterial and viral infections in the setting of other illness: a complex model

The Global Fever-Bacterial/Viral/Noninfectious (GF-B/V/N) classifier provides two probabilities, a measure of bacterial infection or viral infection in the context of nonbacterial/nonviral illness as a control (Supplemental Fig. 1A). Theoretically, this model has the potential for identifying a co-infection if both the probability of bacterial and viral infection were high (Supplemental Fig. 1A). For classification of bacterial infection (bacterial vs. nonbacterial model) the AUROC was 0.92 (Supplemental Fig. 1B), with sensitivity 87.7% (95% CI 79.0–89.8), specificity 84.2% (95% CI 78.2–89.1), and accuracy 85.2% (95% CI 80.6–89.1) (Table 3). For the classification of viral infection (viral vs. nonviral model), AUROC was 0.91 (Supplemental Fig. 1C), with sensitivity 83.7% (95% CI 76.0–89.8), specificity 81.5% (95% CI 74.8–87.1), and accuracy 82.5% (95% CI 77.6–86.7) (Table 3). Similar to the binary model, the GF-B/V/N test demonstrated good performance for a broad range of bacterial and viral pathogens (Supplemental Fig. 1D,E).

Translation of the 2-model GF-B/V/N system to NanoString was exploratory in nature because it only validated the GF-B/V/N test for bacterial and viral illness, evaluating how often bacterial or viral disease was misclassified in the context of nonbacterial/nonviral illness. We measured expression of 33 genes for the bacterial model and 19 for the viral model (Supplemental Table 4B,C). In the validation cohort, the bacterial model had an AUROC of 0.84 (95% CI 0.76–0.93) (Supplemental Fig. 1F), sensitivity of 82.7% (95% CI 69.7–91.8), specificity of 80.4% (95% CI 66.9–90.2), and accuracy of 81.6% (95% CI 72.7–88.5) (Table 3). The viral model had an AUROC of 0.85 (95% CI 0.77–0.93) (Supplemental Fig. 1G), sensitivity of 76.5% (95% CI 62.5–87.2), specificity of 80.8% (95% CI 67.5–90.4), and accuracy of 78.6% (95% CI 69.5–86.1) for viral infection (Table 3). Performance was similar across pathogens (Supplemental Fig. 1H,I), except for a single *Viridans group streptococcus* case.

### Discordant classifications

Discordant cases in the validation cohort were similar between the two classifiers (19 GF-B/V, 19 GF-B/V/N; with overlap of 15 for both models) (Supplemental Table 5). A review of these discordant cases did not identify any pattern with respect to site or pathogen. The relative increased number of Sri Lanka patients was nearly proportional to the total number in the whole cohort. Interestingly, when predictive genes were fixed and the model weights were allowed to vary among the validation cohort, performance improved.

## Discussion

We utilized a 294-participant multinational prospectively enrolled cohort to develop a bacterial versus viral host-response classifier that incorporates LMIC with representation of zoonotic bacteria and arboviruses. While others have utilized publically available data to apply host-response transcriptional classifiers to atypical global infections^33^, this cohort is the largest prospectively enrolled with robust clinical, phenotypic, and adjudication data. Translation of the GF-B/V test to a multiplex gene expression detection platform demonstrated good performance (overall accuracy of 81.6% [95% CI 72.7–88.5]) in independent validation despite different genetic backgrounds, geographies (five countries), and pathogens. For example, a person with a positive GF-B/V NanoString test in the validation cohort was 5-times more likely to have a bacterial infection and 3-times less likely with a negative test. Such a test could provide timely diagnostic reassurance to inform antibiotic use and guide clinical care.

Decreasing morbidity, mortality, and misuse of antimicrobials from infections requires improved diagnosis at the time a patient presents to care. LMIC have decreased laboratory infrastructure, so performing multiple pathogen-based tests is unrealistic. Accurate acute-phase pathogen-based diagnostics do not exist for many bacterial zoonotic infections, such as ricktettsial infections, that require different treatment from antibiotics empirically used for routinely cultivatable organisms. Point-of-care biomarkers commonly utilized in high-resource settings, like C-reactive protein and procalcitonin, have yielded mixed performance in LMIC (e.g., low specificity, poorer performance for bacterial zoonotic pathogens)^27,50,55–57^, and are potentially affected by higher rates of malnutrition, parasitic disease, HIV, and co-infection^58^. Host-response gene expression assays are poised to fill this void^25–27,31–33,59,60^.

Tremendous progress has been made developing host-response diagnostics in HIC in multiple disciplines, including infectious diseases^31,59–61^. Recently, an algorithmic approach utilizing publically available data extended this method to intracellular and atypical pathogens prevalent globally^33^. Rao et al., utilize a co-normalization technique to diminish study variability and batch effects. While the signal for the bacterial versus viral classifier was preserved, the co-normalization technique could potentially reduce biological variability and artificially improve overall performance in a population with increased variability of pathogens and genetic ancestry. Additionally, use of publically available data does not align enrollment criteria or apply an even reference standard. Prospective validation of this promising work will be critical to determine performance in a real world population of global infections.

Taking a different approach, our study utilized existing biorepository specimens of prospectively enrolled patients that meet reliable eligibility criteria and apply a consistent diagnostic reference. This approach preserves biological variability while avoiding potential bias and confounding. Access to participant-level clinical, biologic, and etiologic data allows refinement of the cohort not possible for publically available data. Additionally, the GF-B/V incorporates a significant number of zoonotic bacterial pathogens that are both extracellular (e.g. *Leptospirosis* spp.) and intracellular (e.g. *Ricketsial* spp.) at the model development and validation phase, while other studies have a low percentage of Leptospirosis or other extracellular pathogens representated in LMIC settings^33^.

A binary bacterial versus viral classifier provides a simple approach to identifying bacterial infections, but does not account for other treatable etiologies of suspected infection. Layered diagnostic tests using multiple binary classifiers, like GF-B/V/N, are more generalizable for a global population, and are attractive given the breadth of pathogen diversity and febrile illness globally. Precedent exists for layered transcriptional expression classifiers that incorporate other classes of illness^25,32^. We demonstrate a more complex model can discriminate bacterial from viral infection in an independent validation cohort, but the absence of noninfectious samples in the validation cohort limits full evaluation in a real world population. Thus, we cannot comment on noninfectious illness, but simply on nonbacterial or nonviral disease. However, we demonstrate that misclassification by GF-B/V or GF-B/V/N is largely overlapping, reassuringly demonstrating that incorporating more complexity does not reduce performance in a limited population of bacterial and viral illness. Incorporating multiple models for this and other work has previously been shown and will need to be addressed going forward^62^. While exploratory, a model with this complexity is not available in other published work on global pathogens, such as leptospirosis or rickettsial infection^31,33,63,64^. The composite model could provide a path forward in the complex milleu of global illness.

Host response biomarkers could change clinical practice, but expansion of these diagnostics to LMIC must be inexpensive, easy to operate, and clinically interpretable. Host gene expression diagnostics for non-infectious applications are considered high complexity tests, often run in referral laboratories. However, technical advances have enabled highly multiplexed quantitative, real-time PCR systems that operate in a sample-in, answer-out format with results available in < 1-h^32,36,60^. As simpler host gene expression tests continue to be developed, cost-of-goods and simplicity will be key parameters for their implementation in LMIC settings^65^. Host response-based biomarker panels have also extended to proteomics and metabolomics^64,66,67^, which may be less expensive and amenable to field deployable diagnostics. Progress refining host-response biomarkers in international cohorts must occur alongside technological advances in platform development to allow more rapid translation to LMIC. The results presented here suggest easy translatability of this approach to LMIC.

GF-B/V and GF-B/V/N multi-analyte biomarkers have attractive features, but there are limitations to this study. Translation to a PCR-based detection system revealed lower accuracy in the validation cohort compared to the RNA-seq based classification in the discovery cohort. This could be due to technical differences (e.g., going from RNAseq to NanoString) but is also an expected difference between discovery and validation, the latter of which includes a wider array of infections and variability of illness. Analysis of discordant classifications suggests that genes used in the models have strong predictive power, but that individuals have variability in the amount, or weight, each gene contributes to the model. Consistent with this is the observation that both classifiers had a reduction of performance on pathogens not hightly represented in the discovery cohort (*Viridians group Streptococcus*, non-influenza respiratory viruses, *Coxiella burnetii*). The GF-B/V/N model is constrained by reliance on non-infectious illness as a control rather than being representative of febrile illness globally. Additional limited availability of high confidence noninfectious samples prevented incorporation into the validation cohort, prohibiting validation of the performance of the GF-B/V/N test for nonbacterial/nonviral illness or co-infection. It will be critical for future studies to perform iterations and optimization on expanded cohorts with increased pathogen (e.g. atypical viruses, tuberculosis, malaria, cryptococcus) and host diversity (e.g., a larger cohort of children and immunocompromised hosts) that would be expected to improve model weights, overall performance, and be more representative of febrile illnesses^62^.

We found that novel host transcriptional biomarkers could accurately discriminate diverse bacterial and viral infections, including those endemic in not only high-income temperate regions but also LMIC in the tropics. Translation of these tests to a custom multiplex gene expression platform, such as the NanoString, shows promise for identification of infections in increasingly diverse populations with the future possibility of point-of-care application. Host-response biomarkers to distinguish bacterial from viral infection could improve clinical care and antibiotic stewardship across the globe.

### Supplementary Information

Supplementary Table S1.

Supplementary Table S2.

Supplementary Table S3.

Supplementary Table S4.

Supplementary Table S5.

Supplementary Figure S1.

Supplementary Figure S2.

## Data Availability

All data in this article were generated as part of this work. All RNA sequencing data has been submitted to GEO under accession number GSE211567. NanoString transcripts are included in supplemental information. Token to access GSE211567: obqzkkoarjwpfct.
