# Supplementary material for: Host-response transcriptional biomarkers accurately discriminate bacterial and viral infections of global relevance
Source: Sci Rep. 2023 Dec 18;13:22554. doi: 10.1038/s41598-023-49734-6 (PMC10728077; doi:10.1038/s41598-023-49734-6)
Supplement: Supplementary file 1 — Supplementary Table S1. [file 41598_2023_49734_MOESM1_ESM.pdf]

Supplemental Table 1: Prospective cohort studies used to assemble discovery and validation cohorts. Abbreviations: MAT = microscopic agglutination test, IFA = indirect immunofluorescence assay, CSF = cerebral spinal fluid

Study	Enrollment Dates	Inclusion	Exclusion	Study specific etiology testing
Community Acquired Pneumonia & Sepsis Outcome Diagnostics (CAPSOD, ClinicalTrials.gov NCT00258869)<br><br>Community Acquired Pneumonia and Sepsis Study (CAPSS)	2005-2018	Patient >18 years old with suspected infection AND 2 of the following 4 criteria<br>1. A core temperature of ≥38°C or ≤36°C<br>2. Heart rate of ≥90 beats/min<br>3. Patients > 18 years of age, Respiratory rate of ≥ 20 breaths/min OR PaCO2 of ≤ 32 mm Hg OR Use of Mechanical Ventilation for an acute respiratory process<br>4. White cell count ≥12,000/mm3 or ≤ 4,000/mm3 OR A differential count showing > 10% immature neutrophils	Patient <6 years old.<br><br>Patient is not expected to survive 28 days because of uncorrectable medical condition or active DNR order<br><br>HIV last known CD4 count of <50 mm3<br><br>Acute presence of a cerebral vascular event, active gastrointestinal hemorrhage, acute seizure, drug overdose, burn injury, trauma<br><br>Patient is pregnant	Respiratory Viral Panel
Rapid Diagnostics in Categorizing Acute Lung Infection (RADICAL)	2017-2020	Patient symptoms of respiratory infection AND patient must meet at least 2 of the following 4 criteria<br>1. A core temperature of ≥38°C or ≤36°C<br>2. Patients >18 years of age, Heart rate of ≥ 90 beats/min Patients 13-18 years of age, Heart rate of ≥110 beats/min Patients 6-12 years of age, Heart rate of ≥130 beats/min<br>3. Patients >18 years of age, Respiratory rate of ≥20	Non-respiratory suspected infection requiring antibiotics prior to admission.	Respiratory Viral Panel

		breaths/min Patients 13-18 years of age, Respiratory rate of ≥14 breaths/min Patients 6-12 years of age, Respiratory rate of ≥18 breaths/min OR PaCO2 of ≤32 mm Hg OR Use of Mechanical Ventilation for an acute respiratory process<br>4. Patients > 18 years of age, White cell count ≥12,000/mm3 or ≤4000/mm3 Patients 13-18 years of age, White cell count ≥11,000/mm3 or ≤4,500/mm3 Patients 6-12 years of age, White cell count ≥13,500/mm3 or ≤4,500/mm3 OR A differential count showing > 10% immature neutrophils		
Austere Environments Consortium for Enhanced Sepsis Outcomes (ACESO) -- USA	2014-2016	Adult patients (≥18 years) who presented with a suspected infection (as judged by the attending physician) and who met at least two of three clinical criteria (eg, thermodysregulation, tachypnea, heart rate > 90 bpm) were considered for inclusion.	Known malignancy, chronic renal/hepatic insufficiency, immunosuppressive conditions (not including HIV) or high-dose steroid usage, history of organ transplant, hemodynamically significant gastrointestinal bleeding, anatomic or functional asplenia, acute cardiovascular disease, or general anesthesia or surgery in the past week, patients who were pregnant, had a	Respiratory Viral Panel

			hemoglobin under 6 g/dL, or weighed less than 35kg.	
Austere Environments Consortium for Enhanced Sepsis Outcomes (ACESO) -- Cambodia	2014-Present	Adult patients (≥18 years) who presented with a suspected infection (as judged by the attending physician) and who met at least two of three clinical criteria (eg, thermodysregulation, tachypnea, heart rate > 90 bpm) were considered for inclusion.	Known malignancy, chronic renal/hepatic insufficiency, immunosuppressive conditions (not including HIV) or high-dose steroid usage, history of organ transplant, hemodynamically significant gastrointestinal bleeding, anatomic or functional asplenia, acute cardiovascular disease, or general anesthesia or surgery in the past week, patients who were pregnant, had a hemoglobin under 6 g/dL, or weighed less than 35kg.	Culture: Blood for all, Sputum when available, CSF (if indicated).<br><br>*Burkholderia pseudomallei*<br>-Culture<br>-Modified iSTAT<br><br>Chikungunya virus<br>-acute & conv. serologies<br>-Serum PCR<br><br>Dengue virus<br>-acute & conv. serologies<br>-Serum PCR<br><br>*Haemophilus influenzae*<br>-Culture<br>-Serum PCR<br><br>Hantaviruses<br>-Serum PCR<br><br>Leptospirosis<br>-acute & conv. serologies<br>-Serum PCR<br><br>*Neisseria meningitidis*<br>-Serum PCR<br><br>Orientia tsutsugamushi<br>-acute & conv. Serologies<br>-next genome sequencing<br><br>SFG rickettsioses<br>-acute & conv. serologies<br><br>*Salmonella typhi*

				-Culture<br>-Serum PCR<br><br>*Streptococcus pneumoniae*<br>-Serum PCR<br><br>*Streptococcus suis*<br>-Culture<br>-Serum PCR
Sri Lanka Emerging Acute Febrile and Respiratory Infection Characterization (SEARCh)	2012-2014	Patients ≥1 year of age with documented fever (>38C) at presentation or within 48 hours of hospital admission	Patients with focal bacterial infections such as pneumonia or soft tissue infection.	Dengue<br>-acute & conv. serologies<br>-serum PCR<br>-Dengue virus isolation<br><br>Respiratory Viral Panel<br><br>*Leptospirosis spp.*<br>-acute & conv. serologies<br>-serum PCR<br><br>*Rickettsial spp.*<br>-acute & conv. serologies<br>-serum PCR<br><br>*Coxiella burnetii*<br>-acute & conv. serologies<br>-serum PCR
Tanzania Febrile Illness Study	2012-2014	Patients ≥1 year of age a tympanic temperature ≥38.0°C or a subjective report of fever within the previous 72 h.	None	*Leptospirosis spp.*<br>-acute & conv. serologies<br><br>*Rickettsial spp.*<br>-acute & conv. serologies<br><br>*Brucella spp.*<br>-acute & conv. serologies<br><br>*Coxiella burnetii*<br>-acute & conv. serologies<br><br>Bacterial Blood cultures

				Malarial blood culture or blood smear
Australian pandemic H1N1-2009 study	2009-2010	Adult patients (≥18 years) hospitalized with suspected severe influenza.	None	H1N1-2009 PCR<br>Influenza A and B PCR
