# Supplementary material for: Host-response transcriptional biomarkers accurately discriminate bacterial and viral infections of global relevance
Source: Sci Rep. 2023 Dec 18;13:22554. doi: 10.1038/s41598-023-49734-6 (PMC10728077; doi:10.1038/s41598-023-49734-6)
Supplement: Supplementary file 2 — Supplementary Table S2. [file 41598_2023_49734_MOESM2_ESM.pdf]

Supplemental Table 2: Case definitions for global acute suspected infection cohort adhered to by the adjudication panel. US site adjudication was based on review of full medical records. For international sites information about symptoms and other testing was obtained through the case report forms. *Study performed Respiratory Viral PCR Panel was only performed for USA and Sri Lanka sites because these studies collected nasal swabs.

**Case Definition**	**Case Report Form or Medical Record**	**Microbiological (study)**
Staphylococcus Aureus	Acute infectious symptoms<br>**2 positive blood cultures**<br>Negative microbiology for other pathogens	Negative Respiratory Viral PCR Panel*
Streptococcus<br>--*Streptococcus Pneumoniae*<br>--*Viridians Group Streptococcus*	Acute infectious symptoms<br>**2 positive blood cultures <u>and/or</u> positive *S. pneumonia* Urinary Antigen**<br>Negative microbiology for other pathogens	Negative Respiratory Viral PCR Panel*
Gram Negative Bacteria<br>--*Escherichia Coli*<br>--*Klebsiella Pneumoniae*<br>--*Pseudomonas aeruginosa*	Acute infectious symptoms<br>**2 positive blood cultures**<br>Negative microbiology for other pathogens	Negative Respiratory Viral PCR Panel*
Leptospirosis spp.	Acute infectious symptoms	**4-fold increase in MAT serologies between acute and convalescent serum <u>and/o</u>r serum PCR**<br>Negative Respiratory Viral PCR Panel*<br>Negative for other study performed pathogen testing
Rickettsia spp.	Acute infectious symptoms	**4-fold increase in IFA serologies between acute and convalescent serum <u>and/o</u>r serum PCR**<br>Negative Respiratory Viral PCR Panel*<br>Negative for other study performed pathogen testing
Coxiella burnettii	Acute infectious symptoms	**4-fold increase in IFA serologies between acute and convalescent serum <u>and/o</u>r serum PCR**<br>Negative Respiratory Viral PCR Panel*<br>Negative for other study performed pathogen testing
Brucella spp.	Acute infectious symptoms	**4-fold increase in MAT serologies between acute and convalescent serum**<br>Negative for other study performed pathogen testing.
Burkhoderia pseudomallei	Acute infectious symptoms	**Positive blood culture, sputum culture, and/or Modified iSTAT Antigen**<br>Negative for other study performed pathogen testing
Influenza A or B	Acute infectious symptoms<br>Negative for other pathogens	**Positive Respiratory Viral PCR (clinical or study performed).**<br>Negative for other study performed pathogen testing.
Dengue	Acute infectious symptoms	**Isolation of Dengue virus**<br>**Consistent PCR and acute and convalescent serologies**

		Negative for other study performed pathogen testing
Other Respiratory Viruses<br>- Respiratory Syncytial Virus<br>- Human Rhinovirus<br>- Human Metapneumovirus<br>- Parainfluenza Virus 1-4	Acute infectious symptoms<br>Negative for other pathogens	**Positive Respiratory Viral PCR Panel (clinical or study performed)**<br>Negative for other study performed pathogen testing
Noninfectious Syndrome	**High confidence noninfectious diagnosis AND Negative testing for bacterial and viral pathogens AND consistent clinical information (radiology, clinical course, etc.)**	Negative Respiratory Viral PCR Panel
