# Supplementary material for: Host-response transcriptional biomarkers accurately discriminate bacterial and viral infections of global relevance
Source: Sci Rep. 2023 Dec 18;13:22554. doi: 10.1038/s41598-023-49734-6 (PMC10728077; doi:10.1038/s41598-023-49734-6)
Supplement: Supplementary file 3 — Supplementary Table S3. [file 41598_2023_49734_MOESM3_ESM.pdf]

Supplementary Table 3: We expanded the GF-B/V and GF-B/V/N models to include correlated transcripts that can substitute for one another to predict bacterial or viral disease. Predictive transcripts (263) were included on the NanoString gene expression assay to allow optimization.

gene	accession	gene	accession	gene	Accession
LRRC3	NM_030891.3	ATG2A	NM_015104.2	C1orf228	NM_001145636.1
CD40	NM_001250.4	XIST	NR_001564.2	LOXL1	XM_011521555.1
WDR27	NM_182552.3	ROM1	NM_000327.3	PDGFA	NM_002607.5
ARPC3	NM_005719.2	YWHAB	NM_139323.3	ATP1B2	NM_001678.3
TMEM255A	NM_017938.3	NEXN	NM_144573.3	JUP	NM_002230.2
SMARCD3	NM_001003801.1	EREG	NM_001432.2	CEACAM8	NM_001816.3
HSH2D	NM_032855.2	KLF14	NM_138693.2	HLA.DRB4	NM_021983.4
USP18	NM_017414.3	ANKRD22	NM_144590.2	CIB2	NM_006383.3
PEX6	NM_000287.3	DLEU7.AS1	NR_046551.1	MRPS18B	NM_014046.3
POLR2F	NM_021974.3	SNHG9	NR_003142.2	BTN2A2	NM_181531.2
HLA.DRB3	NM_022555.3	FAM20A	XM_006721959.2	KPNB1	NM_002265.4
NDUFB1	NM_004545.3	MTMR1	NM_003828.2	SFN	NM_006142.3
UNK	NM_001080419.2	TMEM165	NM_018475.4	MYL6B	NM_001199629.1
RTCB	NM_014306.4	IRF9	NM_006084.4	PIMREG	NM_019013.1
SBDS	NM_016038.2	PLAC8	NM_016619.2	H1F0	NM_005318.3
KIDINS220	NM_020738.2	PAX5	NM_016734.1	LGSN	NM_001143940.1
ZNF335	NM_022095.3	BMX	NM_001721.6	TUBB1	NM_030773.3
C1QC	NM_001114101.1	SLC39A8	NM_022154.5	DSP	NM_001008844.1
AC009948.1	NR_110204.1	SSTR3	NM_001051.3	LRRN3	NM_001099660.1
TSTA3	NM_003313.3	HES4	NM_001142467.1	PGA4	NM_001079808.3
RALY.AS1	NR_109886.1	KRIT1	NM_004912.3	HIST2H2BF	NM_001024599.3
HIRA	NM_003325.3	C22orf39	NM_173793.4	MIR142	NR_029683.1
EBI3	NM_005755.2	STOML1	NM_004809.4	GIN1	NM_017676.2
PLSCR1	NM_021105.2	IFT20	NM_001267774.1	AC114936.1	NR_003615.2
HLA.DQB1	NM_002123.4	GLIPR1	NM_006851.2	CCR1	NM_001295.2
TNFAIP6	NM_007115.2	SLC18B1	NM_052831.2	ZNF83	NM_001277946.1
EIF2AK2	NM_002759.1	SP100	NM_001206704.1	ENDOD1	NM_015036.2
PSMD6	NM_001271779.1	PLEKHA7	NM_175058.4	HYAL3	NM_001200029.1
ORAI2	NM_001271819.1	NECAB1	NM_022351.4	DERL2	NM_016041.3
NKAIN1	NM_024522.2	IFI35	NM_005533.3	GLUD1	NM_005271.2
CXCL9	NM_002416.1	TTC26	NM_001144920.2	TSC22D1	NM_183422.3
CPNE1	NM_001198863.1	PATL2	NM_001145112.1	SLAMF8	NM_020125.2
ENTPD5	NM_001249.2	AIM2	NM_004833.1	FAP	NM_004460.2
ARHGAP12	NM_001270698.1	LINC01781	NR_125942.1	CDKN1A	NM_000389.2
EPHB2	NM_004442.6	JUND	NM_005354.5	LGMN	NM_001008530.2
S100A8	NM_001319198.1	LINC00211	NR_110011.1	TRIM22	NM_006074.4
TTC21A	NM_145755.2	HLA.DRB1	NM_002124.3	ARPIN	NM_182616.3
TRMT13	NM_019083.2	ZNF189	NM_197977.2	CCL2	NM_002982.3
ANKRD45	NM_198493.2	SH3BP1	NM_018957.3	ZNF331	NM_001079906.1
CLDN5	NM_001130861.1	PLVAP	NM_031310.1	PPM1N	NM_001080401.1
SERTAD2	NM_014755.1	MC1R	NM_002386.2	LY6E	NM_001127213.1
ALPL	NM_000478.4	NAA38	NM_032356.3	GGTLC2	NM_199127.2
ADAMTS17	NM_139057.2	FBXO6	NM_018438.5	CAMK1	NM_003656.3
ANKRD11	NM_001256182.1	AXL	NM_021913.2	PADI2	NM_007365.2
MST1	NM_020998.3	OSBP2	NM_030758.3	ENC1	NM_003633.2
PTRHD1	NM_001013663.1	FAM104B	NM_001166704.1	S100A12	NM_005621.1
TRIM69	NM_080745.4	AGRN	NM_198576.3	HSPB9	NM_033194.3
MT1G	NM_005950.2	IFIT1B	NM_001010987.2	RPS2P32	NR_026676.1
TNFSF8	NM_001244.3	ZNF577	NM_032679.2	HESX1	NM_003865.2
SLC51A	NM_152672.5	IFITM1	NM_003641.3	PPP6R3	NM_001164162.2
SLAMF7	NM_021181.4	UQCRH	NM_006004.2	CCDC152	NM_001134848.1

gene	Accession	gene	accession	gene	Accession
AC026304.1	NR_034032.1	HACD1	NM_014241.3	SULT1C4	NM_001321770.1
ISL2	NM_145805.1	CD44	NM_001001392.1	CA4	NM_000717.4
NCR1	NM_004829.6	FPR3	NM_002030.3	ZBTB8OS	NM_178547.4
CYSLTR2	NM_020377.2	EPHB1	NM_004441.3	NQO2	NM_000904.3
YBEY	NM_001006114.2	SH3YL1	NM_001159597.1	HEATR1	NM_018072.5
SLC9A8	NM_015266.3	SPRED2	NM_001128210.1	ZMYND15	NM_032265.1
HMGB3	NM_005342.2	TIMM8B	NR_028383.1	ERICH3	NM_001002912.4
C1QA	NM_015991.2	DDX3Y	NR_136717.1	SIGLEC1	NM_023068.3
CD177	NM_020406.4	OTOF	NM_004802.3		
BTF3	NM_001037637.1	C4A	NM_007293.2		
PGAP1	NM_024989.3	CDK5RAP2	NM_001011649.1		
PYGL	NM_002863.3	RPL28	NM_000991.4		
CHI3L1	NM_001276.2	FFAR3	NM_005304.2		
BX284668.5	NR_135059.1	TGIF1	NM_003244.2		
IFI27	NM_005532.3	KANSL2	NM_017822.3		
RABGAP1L	NM_001035230.2	NOV	NM_002514.2		
ADGRE3	NM_032571.5	GIMAP6	NM_024711.5		
ZSWIM8.AS1	NR_038357.1	EXOC7	NM_001145298.3		
KREMEN1	NM_001039570.1	GPA33	NM_005814.1		
SRBD1	NM_018079.4	4.Sep	NM_001198713.1		
CD300LD	NM_001115152.1	OASL	NM_198213.2		
SIGLEC10	NM_001171158.1	ANAPC15	NM_001278486.1		
GPR84	NM_020370.2	RNF43	NM_017763.4		
KIR3DS1	NM_001083539.1	ICAM4	NM_001039132.2		
IQCE	NM_152558.4	TES	NM_015641.3		
PGLYRP1	NM_005091.3	ZDHHC19	NM_001039617.1		
MICAL2	NM_001282663.1	TRAK1	NM_014965.3		
CKLF.CMTM1	NM_001202509.2	IFIT1	NM_001548.3		
RETREG3	NM_178126.4	NCR3LG1	NM_001202439.2		
SCAPER	NM_001145923.1	DEFA1B	NM_001042500.1		
CCL8	NM_005623.2	PRF1	NM_001083116.2		
GGT1	NM_001032364.2	SERTAD3	NM_203344.1		
EMID1	NM_133455.3	TNFSF10	NM_003810.4		
DZIP1L	NM_001170538.1	HIKESHI	NM_016401.4		
MIAT	NR_003491.2	HERC6	NM_001165136.1		
DBF4B	NR_036623.1	ADCY9	NM_001116.3		
HERC1	NM_003922.3	IL4I1	NM_152899.1		
DEFA1	NM_004084.2	CBX7	NM_175709.3		
CDK18	NM_002596.3	MSL1	NM_001012241.1		
CD59	NM_000611.4	ADGRE1	NM_001974.3		
ZSCAN12	NR_136510.1	ITPR3	NM_002224.3		
ADAMTSL4	NM_019032.5	RCVRN	NM_002903.2		
CFAP126	NM_001013625.2	PTPRS	NM_002850.3		
FAM13A	NM_001265579.1	GNG7	NM_052847.2		
LPIN2	NM_014646.2	PSPH	XM_005271773.1		
PPCDC	NM_021823.3	TP53INP2	NM_021202.1		
LINC02218	NR_134270.1	C1QB	NM_000491.3		
CACNA1E	NM_000721.3	NDUFB6	NM_002493.5		
YTHDF3.AS1	NR_102684.1	IL1RN	NM_173843.2		
RUNX1	NM_001001890.2	ERC1	NR_027946.2		
DIP2C	NM_014974.2	KLHDC8B	NM_173546.2		
