# Supplementary material for: Host-response transcriptional biomarkers accurately discriminate bacterial and viral infections of global relevance
Source: Sci Rep. 2023 Dec 18;13:22554. doi: 10.1038/s41598-023-49734-6 (PMC10728077; doi:10.1038/s41598-023-49734-6)
Supplement: Supplementary file 4 — Supplementary Table S4. [file 41598_2023_49734_MOESM4_ESM.pdf]

Supplemental Table 4: Genes used for (A) GF-B/V model and GF-B/V/N models: (B) bacterial vs nonbacterial, (C) viral vs nonviral

A. GF-B/V model

gene	coefficient
SBDS	-0.2039
TNFAIP6	0.032258
SLAMF7	0.017245
SLC39A8	0.424612
LINC00211	-0.13563
SH3BP1	-0.27372
MC1R	0.051549
PDGFA	-0.03023
CIB2	-0.13634
MRPS18B	-0.67827
ZNF83	-0.10735
SLAMF8	0.481558
CDKN1A	-0.30245
LGMN	-0.03615
CCL2	-0.42712
CCDC152	-0.08007
CHI3L1	-0.03487
IFI27	-0.05835
CD300LD	0.044026
DZIP1L	-0.14345
CACNA1E	0.195847
YTHDF3.AS1	0.490684
FPR3	-0.09259
ADGRE1	0.128859
TP53INP2	-0.3112
C1QB	0.742775
KLHDC8B	-0.29363

B. GF-B/V/N model: bacterial vs. nonbacterial

gene	coefficient
SBDS	-0.22835
TNFAIP6	0.069537
CLDN5	-0.0723
SLAMF7	0.010827
SLC39A8	0.448561
LINC00211	-0.20356
SH3BP1	-0.36912
MC1R	0.132077
PDGFA	-0.09077

CIB2	-0.12449
MRPS18B	-0.73713
ZNF83	-0.14596
SLAMF8	0.488592
CDKN1A	-0.28538
LGMN	-0.02387
CCL2	-0.43832
ZNF331	-0.00278
CCDC152	-0.14905
CHI3L1	-0.0296
IFI27	-0.06545
CD300LD	0.088185
KIR3DS1	-0.00574
IQCE	0.03968
DZIP1L	-0.20136
CACNA1E	0.17706
YTHDF3.AS1	0.549918
FPR3	-0.1591
ADGRE1	0.206039
RCVRN	0.040174
PSPH	0.002308
TP53INP2	-0.33318
C1QB	0.762756
KLHDC8B	-0.29528

C. GF-B/V/N – viral vs. nonviral

gene	coefficient
EIF2AK2	0.109558
TTC21A	0.095119
PTRHD1	-0.08043
NEXN	0.258914
PDGFA	0.017522
ZNF83	0.162538
SLAMF8	-0.12493
CCL2	0.047256
IFI27	1.316791
CD300LD	-0.1908
KIR3DS1	0.043894
IQCE	-0.13931
DBF4B	0.154563
YTHDF3.AS1	-0.30056
HACD1	-0.07536
ADGRE1	-0.08038
PSPH	-0.09827

C1QB	-0.45122
KLHDC8B	0.056937
