# Supplementary material for: Host-response transcriptional biomarkers accurately discriminate bacterial and viral infections of global relevance
Source: Sci Rep. 2023 Dec 18;13:22554. doi: 10.1038/s41598-023-49734-6 (PMC10728077; doi:10.1038/s41598-023-49734-6)
Supplement: Supplementary file 5 — Supplementary Table S5. [file 41598_2023_49734_MOESM5_ESM.pdf]

Supplemental Table 5: Nineteen cases are discordant for each of the models (GF-B/V, GF-B/V/N), with fifteen overlapping. The adjudicated reference standard phenotype is represented in the second column. The predicted phenotype by GF-B/V (column 3) or GF-B/V/N (column 4) are listed. Pathogen identification and clinical information are listed in column 4-6. While all cases underwent testing to determine positive etiology, this was only exhaustive to rule-out co-infection in a subset. Abbreviations: CT = computerized tomography scan, CXR = chest x-ray, ED = Emergency Department, HIV = Human Immunodeficiency Virus.

Site	Reference Standard	GF-B/V prediction	GF-B/V/N prediction	Pathogen	Days of illness	Clinical factors:
Cambodia	BACTERIAL	VIRAL	BACTERIAL	*Burkholderi pseudomallie*	--	HIV negative.
Cambodia	BACTERIAL	VIRAL	VIRAL	*Burkholderi pseudomallie*	--	HIV negative.
USA	BACTERIAL	VIRAL	VIRAL	*Viridians group Streptococcu*s	1	Cocaine intoxication, severe sepsis, endocarditis and bacteremia. HIV negative.
USA	VIRAL	BACTERIAL	BACTERIAL	Respiratory Syncytial Virus	10	Elderly, hospitalized, CT Abdomen/pelvis & CXR negative, no blood cultures drawn. HIV negative.
USA	VIRAL	VIRAL	BACTERIAL	Influenza A / B	6	Hospitalized w/ chills, congestion, sore throat, cough, shortness of breath. CXR w/ unilateral streaky opacity (not consolidative) but thought related to viral illness by adjudicator. No blood or sputum cultures obtained. HIV negative.
USA	VIRAL	VIRAL	NONINFECTIOUS	Human Metapneumovirus	3	Discharged from ED, typical viral syndrome. HIV negative.
Sri Lanka	VIRAL	VIRAL	BACTERIAL	Dengue	4	symptoms 1 day, fever 4 days: joint pain, muscle pain, lost appetite
Sri Lanka	VIRAL	BACTERIAL	BACTERIAL	Dengue	19	5 days fever, respiratory symptoms, poor appetites x10 days with >2 weeks feeling poorly
Sri Lanka	VIRAL	VIRAL	BACTERIAL	Human Rhinovirus / Enterovirus	7	negative CXR, Mild fever, respiratory symptoms, headache, muscle and joint pain
Sri Lanka	VIRAL	BACTERIAL	BACTERIAL	Dengue	3	fever, muscle & joint pain, headache
Sri Lanka	VIRAL	BACTERIAL	VIRAL	Dengue	3	CXR with effusion, fever, cough, bleeding
Sri Lanka	VIRAL	BACTERIAL	BACTERIAL	Dengue	10	CXR with effusion, fever, cough, shortness of breath, muscle & joint pain
Sri Lanka	VIRAL	BACTERIAL	BACTERIAL	Dengue	11	CXR negative, fever, fatigue, poor appetite, muscle pain
Sri Lanka	BACTERIAL	VIRAL	BACTERIAL	Rickettsia spp.	1	fever, pleuritic pain, fatigue, abdominal pain

Sri Lanka	VIRAL	BACTERIAL	BACTERIAL	Human Rhinovirus / Enterovirus	2	fever, headache, minimal respiratory symptoms
Sri Lanka	BACTERIAL	VIRAL	VIRAL	*Leptospiria spp.*	5	fever, headache, joint pain, poor appetite, upper respiratory tract symptoms
Sri Lanka	BACTERIAL	VIRAL	VIRAL	*Leptospira spp.*	14	fever, headache, poor appetite, muscle pain
Sri Lanka	BACTERIAL	VIRAL	VIRAL	*Rickettsia spp.*	5	fever, cough, poor appetite
Sri Lanka	VIRAL	BACTERIAL	BACTERIAL	Human Rhinovirus / Enterovirus	2	fever, cough, runny nose, shortness of breath
Tanzania	BACTERIAL	VIRAL	NONINFECTIOUS	*Rickettsia spp.*	4	fever, cough, joint pain
Tanzania	BACTERIAL	VIRAL	BACTERIAL	*Coxiella burnetii*	3	fever, cough, dyspnea, headache, rigors, night sweats, HIV infected.
Tanzania	BACTERIAL	VIRAL	VIRAL	*Coxiella burnetii*	3	fever, illness duration 3 days, cough, dyspnea, headache, joint pain, rigors, night sweats. HIV negative.
Tanzania	BACTERIAL	VIRAL	VIRAL	*Coxiella burnetii*	21	Prolonged fever, worsening dyspnea, joint pain, rigors, nights sweats, diarrhea, abdominal pain. HIV negative.
